# Production and characterization of bio-oils from fast pyrolysis of tobacco processing wastes in an ablative reactor under vacuum

**DOI:** 10.1371/journal.pone.0254485

**Published:** 2021-07-16

**Authors:** Nattawut Khuenkaeo, Sanphawat Phromphithak, Thossaporn Onsree, Salman Raza Naqvi, Nakorn Tippayawong

**Affiliations:** 1 Faculty of Engineering, Department of Mechanical Engineering, Chiang Mai University, Chiang Mai, Thailand; 2 Department of Chemical Engineering, National University of Sciences and Technology, Islamabad, Pakistan; Tocklai Tea Research Institute, INDIA

## Abstract

Application of advanced pyrolysis processes to agricultural waste for liquid production is gaining great attention, especially when it is applied to an economic crop like tobacco. In this work, tobacco residues were pyrolyzed in an ablative reactor under vacuum. The maximum bio-oil yield of 55% w/w was obtained at 600°C with a particle size of 10 mm at a blade rotation speed of 10 rpm. The physical properties of the products showed that the oil produced was of high quality with high carbon, hydrogen, and calorific value. Two-dimensional gas chromatography/time-of-flight mass spectrometric analysis results indicated that the oils were complex mixtures of alkanes, benzene derivative groups, and nitrogen-containing compounds. In addition, ^13^C NMR results confirmed that long aliphatic chain alkanes were evident. The alkanes were likely converted from furans that were decomposed from hemicelluloses. Ablative pyrolysis under vacuum proved to be a promising option for generating useful amount of bio-oils from tobacco residues.

## 1. Introduction

Due to the depletion of fossil fuel supplies, along with major environmental concerns, renewable energy sources such as solar, wind, hydro, geothermal, and biomass are becoming enormously important. Among these sources, biomass has drawn attention because of its possible transformation into valuable oil products. Agro-waste is one of the most significantly exploited renewable energy sources; for example, it is used to produce fuels and valuable chemicals [1]. In the north of Thailand, tobacco is a major economic crop, and harvesting and processing it generates large amounts of waste, which is toxic if managed poorly. However, this waste can be used to generate valuable and useful products via pyrolysis [2].

Pyrolysis is a thermal cracking process for biomass feedstock without or with a limited supply of oxygen. It usually operates in a temperature range of 300–700°C. Liquid oil, solid char, and gas are three major products of the pyrolysis process [3–6]. A thermal reactor that provides heating is a major part of the process. Ablation is about pressing a material against hot surface with high relative motion. It is a unique rapid heating technique that enables direct heat transfer between relatively large sized biomass and heat source, consequently the rigorous reduction in feed size is not required. Ideally, the pyrolytic volatiles generated need to be rapidly removed from the reactor and quenched, possibly in a condensing system, to avoid secondary cracking reactions and repolymerization, and formed bio-oil [5–7]. A vacuum pyrolysis system may be adopted without the need of carrier gas to generate valuable reactive intermediates [8,9]. The relative yield and characteristics of liquid product depends on the operating parameters and the properties of the biomass.

Biomass pyrolysis produces liquid bio-oil, which is a source of biochemicals and biofuels. Several works have pyrolyzed tobacco waste to obtain high-value products such as biochar [10,11], which investigated the effects of pyrolysis conditions on solid residue and the physiochemical properties of the char. Some researchers have investigated thermal decomposition [12,13], studying the potential of pyrolysis for the valorization of tobacco using thermogravimetric (TG) analysis and TG-Fourier transform infrared (FTIR) spectroscopic techniques to investigate the effect of temperature and heating rate on the products [14,15]. It is well known that slow pyrolysis results in high char yield, whereas high yields of liquid oil are always obtained from fast pyrolysis. Pütün et al. [16] investigated yields and chemical composition between slow and fast pyrolysis. They found that not only was the type of biomass significant to the distribution of the yield and characterization of the liquid oil but also the operating conditions were crucial. Gozan et al. [17] studied the effect of high temperatures of 500 to 700°C and found that the optimum yield of liquid oil was obtained at 600°C. Cardoso et al. [18] investigated the effect of temperatures (400–700°C) and additives (ZnCl_2_ and MgCl_2_) on the distribution of chemical compounds in the liquid oil from the pyrolytic reaction. They found that the liquid oil could be used to produce a fuel with good ignition.

A one-dimensional gas chromatography–mass spectrometry (GC-MS) is usually employed to analyze chemical compounds in the liquid oil from pyrolytic processes and the final product. Further insight into chemical compounds could be achieved by adapting the py-GC-MS technique [19], TG-FTIR, and TG-MS [20]. Yan et al. [21] studied the chemical compounds extracted from tobacco leaves and stems. They reported that most aromatic components were recovered at temperatures below 350°C and fragmented into small molecules with increasing temperature due to secondary decomposition. Other specific chemical compounds such as nitrogen-containing compounds [22], nicotine [23,24], and phenols [25,26] have been studied. One dimensional GC has been used for many years; however, there remain minor shortcomings in obtaining the best compound separation using this technique. Increasing the resolution and detectability of liquid oil could be obtained using higher-dimensional GC techniques [27], allowing for the detection of a higher number of compounds. A 2-dimensional, GCxGC/TOF MS analysis is believed to be more efficient in identifying more organic compounds in the organic fraction than traditional GC-MS. Even though there have been several studies on tobacco bio-oils, 2-dimensional GC analysis of bio-oils is still scarce.

In this work, the yield and characterization of bio-oil, including aqueous and organic phases, was studied. The effects of temperature, blade rotation speed, and particle size through the ablative reactor were investigated. The elemental and chemical compositions of the organic phase of the liquid oils were analyzed by GCxGC/TOF MS and nuclear magnetic resonance (NMR).

## 2. Experimental

### 2.1 Sample materials

**Fig 1:**
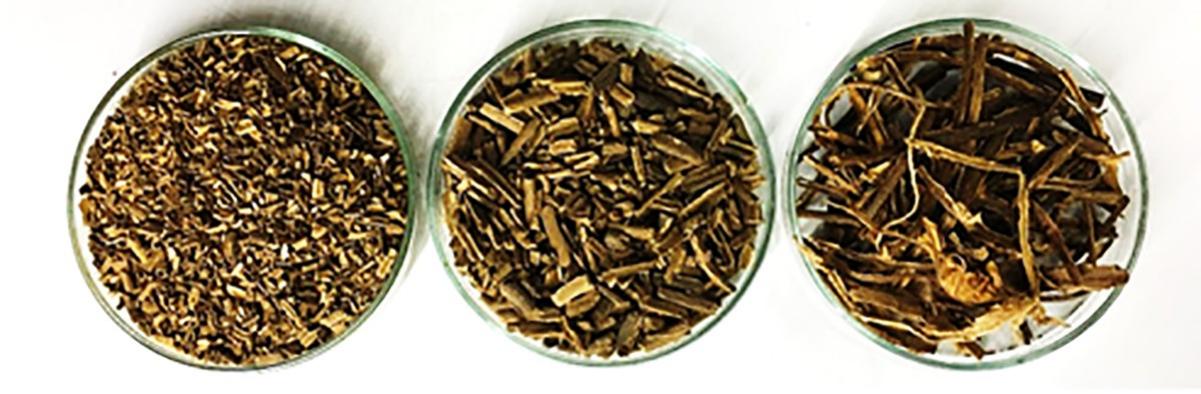
Tobacco residues with particle sizes 0.5 cm, 1 cm, and 3–5 cm.

Residues from tobacco harvested and processed at the Tobacco Authority of Thailand ‘s Denchai redrying plant were used as the raw material in this work. They contained 8.2–15.7% celluloses, 7.5–7.6% hemicelluloses, and 7.1–9.1% lignin [2]. The samples were cut in lengths of 0.5 cm, 1 cm, and 3–5 cm, as shown Fig 1, dried at 60°C for 8 h, and stored in Ziploc bags. This feedstock was used to generate the oil with pyrolysis.

### 2.2 Experimental procedure

About 50 g of dried feedstock was introduced into a rotating blade ablative reactor under vacuum at approximately −10.7 kPa (gauge) for each experiment. The pyrolysis setup was adapted from that used in our previous work [2]. In the pyrolytic chamber, the feedstock was heated during effective ablation with four asymmetric blades, which were rotated and pressed against a hot plate. The pyrolytic temperatures varied: 450, 500, 550, and 600°C, while the residence time was fixed at 10 min. During the biomass ablation, volatiles released from the biomass decomposition could be extracted from the reactor; therefore, secondary reactions of the volatiles were prevented. The speed of the blades was varied at 1, 8, and 10 rpm. The pyrolysis liquid products consisting of the organic and aqueous phases were obtained using a dry ice condenser. The yield measurements were replicated at least three times for each condition in order to guarantee reproducibility, and the results were averaged. The collected liquid was weighed after removing the dry ice. The aqueous phase was separated from the organic phase, while the organic phase that adhered to the condenser was dissolved with acetone. Then, both fractions of the oil were stored in enclosed glass bottles in the dark at 0–2°C to avoid any change in the physical properties or chemical composition. The organic phase was further analyzed. The percentage of the oil products was calculated from the material balance relative to the mass of the sample feed.

$$Y_{L}=\frac{M_{L}}{M_{F}}\times 100$$ (1)

where M_L_ and M_F_ are the masses of the liquid bio-oil product and the biomass feed, respectively, and Y_L_ is the yield of the liquid bio-oil.

### 2.3 Feedstock and product analysis

The samples and products were analyzed for proximate and ultimate composition as well as calorific value. In the proximate analysis, the moisture was determined according to ASTM E871. The ash content was inferred following the standard ASTM D1105. The volatile matter was determined using an oven at 980 ± 10 ^o^C, and approximately 1 g of the sample was placed inside of the preheated oven for 10 min. Finally, the sample was removed from the oven, cooled in a desiccator, and the residual masses were determined. Proximate analyses were performed in triplicate for each sample. The ultimate analysis for carbon, hydrogen, nitrogen, and oxygen was carried out using the ASTM D5373 standard, and the sulfur content was determined using ASTM D4239.

^1^H and ^13^C NMR were used to analyze the functional groups of the bio-oil samples in deuterated acetone with a Bruker 400 UltraShield NMR that scanned the bio-oil samples at each operating temperature. The ^1^H NMR was operated at 400 MHz and 21°C. The result was represented with a spectral width of 8278 Hz and a resolution of 0.126 Hz. The ^13^C NMR was operated at 100 MHz and 22°C, and also used the ^1^H-BB probe of 5 mm BBI with a spectral width of 23980 Hz and a resolution of 0.366 Hz. The NMR spectra were evaluated in MestreNova and were accurately standardized to the acetone reference peak. The chemical shifts scanned during NMR were 0–16 ppm for ^1^H and 0–220 ppm for ^13^C. The peak results were integrated and then normalized to the total area for representation.

The GCxGC/TOF-MS instrument used consists of a liquid nitrogen quad-jet modulator and a CTC Combipal autosampler, operated in electron ionization mode with an energy of 70 eV, mass acquisition in the range of 50–550 m/z at 100Hz, and a detector voltage of 1706 V. The transfer line and injector were kept at 553.15 K, while the ion source was kept at 523.15 K. Two conventional columns were used: a DB-5 (5% phenyl and 95% dimethylpolysiloxane) with a length of 60 m, an internal diameter of 0.25 mm, and film thickness of 0.1 μm; and DB-17MS (50% phenyl and 50% dimethylpolysiloxane) with a length of 2.15, an internal diameter of 0.18 mm, and film thickness of 0.18 μm. The DB-5 column started at 40°C for 4 min and heated at a rate of 4°C to 280°C, remaining at this value for 15 min. The second column was maintained at 10°C above the temperature of the first column. The modulation period was 7 s, and the hot pulse was 40% of the modulation period.

The relative area percent for each chromatographic peak was employed as a semi-quantitative approach to evaluating the contribution of each compound area to the total area and for comparison between the different pyrolytic temperatures of the bio-oil samples. The sum of all peak areas was considered to be 100% of the sample. The peak areas related to solvent and column bleed were ignored.

## 3. Results and discussion

### 3.1 Product distribution and yields

**Fig 2:**
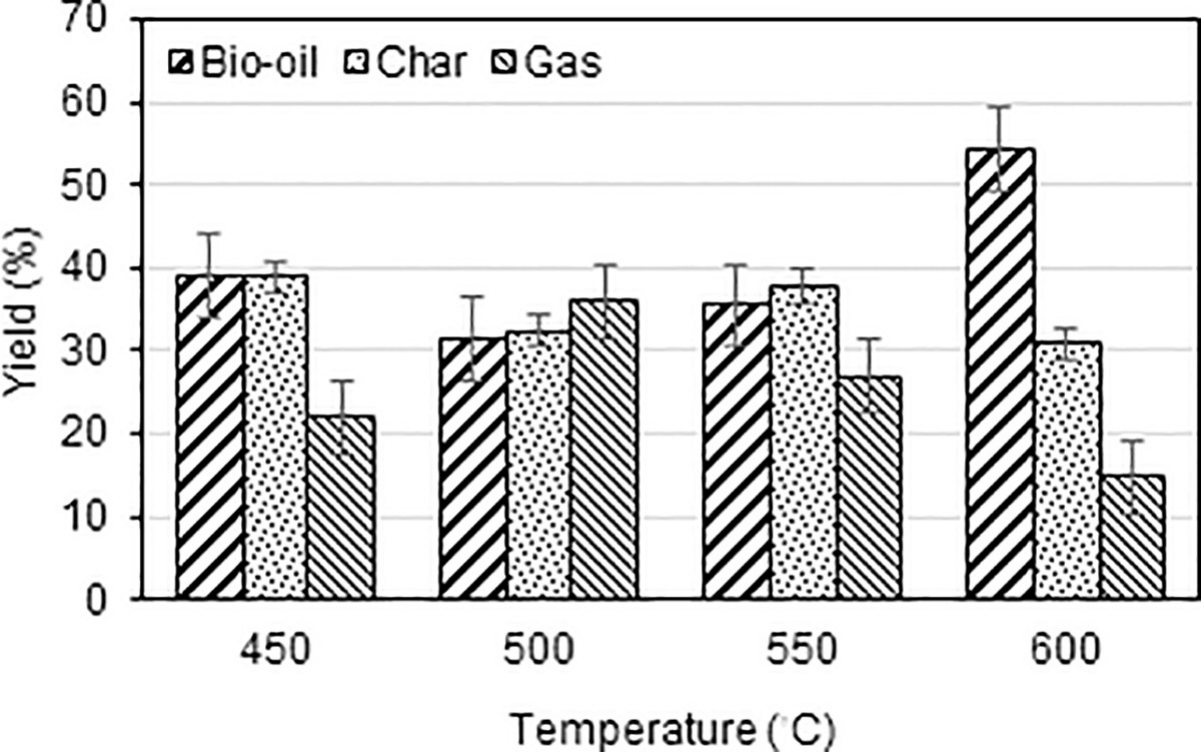
Product distribution at a particle size of 1 cm and blade speed of 10 rpm.

During pyrolysis, many factors contribute to the yield and character of the products. From the configuration of the reactor, three parameters associated with the thermal degradation of raw biomass are the temperature, particle size, and speed of the rotating blade. Fig 2 shows the distribution and yield of the pyrolysis products. The highest bio-oil yield of 54.5% w/w was obtained at 600°C, in similar range to that reported in previous research [28], and the highest char yield was received at 450°C. As anticipated, lowering the temperature tended to result in higher char yield [29,30]. Nonetheless, the char yields at 500 and 550°C might have been affected by other factors, such as particle size and blade speed, that directly relate to heat transfer.

**Fig 3:**
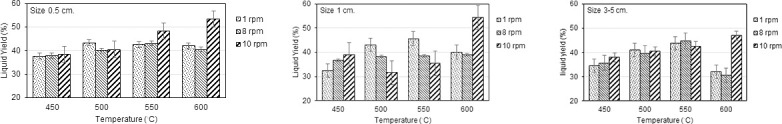
The effect of particle sizes of (a) 0.5 cm, (b) 1 cm, and (c) 3–5 cm on the liquid yields at varying temperatures and rotation speeds.

As far as the particle size was concerned, the results from the smallest particle size (0.5 cm) indicate that the oil yield was affected by varying temperature and rotating speed, as shown in Fig 3(A). At low and moderate pyrolytic temperatures, the temperature change did not affect the oil yield. At a relatively high temperature of 550°C, higher speeds result in higher yields, from 42% w/w at 1 rpm to about 48% w/w at 10 rpm. A similar trend was observed at 600°C when 40% oil yield was improved to 55% w/w when the speed was increased from 1 to 10 rpm.

From Fig 3(B), with the moderate particle size of 1 cm, at 450°C, the oil yield increases with increasing rotating speed from approximately 32% to 37% and 39% w/w with rotating speeds of 1 to 8 and 10 rpm, respectively. The oil yields at moderate temperatures, 500 and 550°C, have opposite trends, such that the oil yield decreases with increasing speed. At the highest temperature (600°C), the oil yields were improved at all speeds, reaching a maximum of 54.4% w/w yield at the highest speed.

For the largest particle size considered, Fig 3(C) shows a slight increase in the oil yields with increasing speed at 450–550°C. The yields were similar to those obtained from other particle sizes at similar temperatures. At 600°C, the oil yields were lower at rotation speeds of 1 and 8 rpm, but reached a maximum at the highest speed considered.

### 3.2 Proximate and ultimate analysis of the raw biomass and main products

Proximate results, including fixed carbon and elemental components, as well as the estimate of the heating value of the raw biomass, are shown in Table 1. Ultimate analysis results of the raw biomass and the main pyrolysis products are also included. The raw biomass appears to have high volatile matter (59%) and ash (21%) contents. This volatile matter content was slightly lower, and the ash content was much higher than those reported in other works [21,29,31]. For elemental analysis, the C and H concentrations of the bio-oil were 75.1% and 9.2%, respectively, whereas the char values were 59.9% and 3.2%, respectively. Both products showed a higher carbon content than the raw tobacco residues. Moreover, the oxygen content in the oil (11.8%) was lower than in the original biomass feedstock (48.1%) and the char (33.6%). This implied that the bio-oil was likely to be a good liquid fuel. However, the amounts of nitrogen and sulfur present in the oil were high, which poses a challenge for the upgrading process to obtain high-quality biofuels or valuable chemicals.

**Table 1:** Proximate* and ultimate** analysis results of raw tobacco residues and the main pyrolysis products.

	Moisture (%)	VM (%)	FC (%)	Ash (%)	C (%)	H (%)	O (%)	N (%)	S (%)	HHV (MJ/kg)	Ref
Raw	13.01	59.45	6.82	20.72	41.74	5.00	48.05	5.12	0.09	14.53	This work
Raw	12.30	72.75	1.69	13.26	42.39	6.49	48.48	2.23	0.41	14.98	[31]
Raw	10.80	68.54	11.78	8.88	46.40	5.96	45.56	2.08	n/a	n/a	[21]
Char	n/a	n/a	n/a	n/a	59.92	3.23	33.60	2.07	1.18	13.25	This work
Bio-oil	n/a	n/a	n/a	n/a	75.07	9.15	11.84	3.80	0.14	30.05	This work

* as-received basis

** dry ash-free basis.

### 3.3 Chemical composition of the bio-oil

#### 3.3.1 NMR analysis

**Fig 4:**
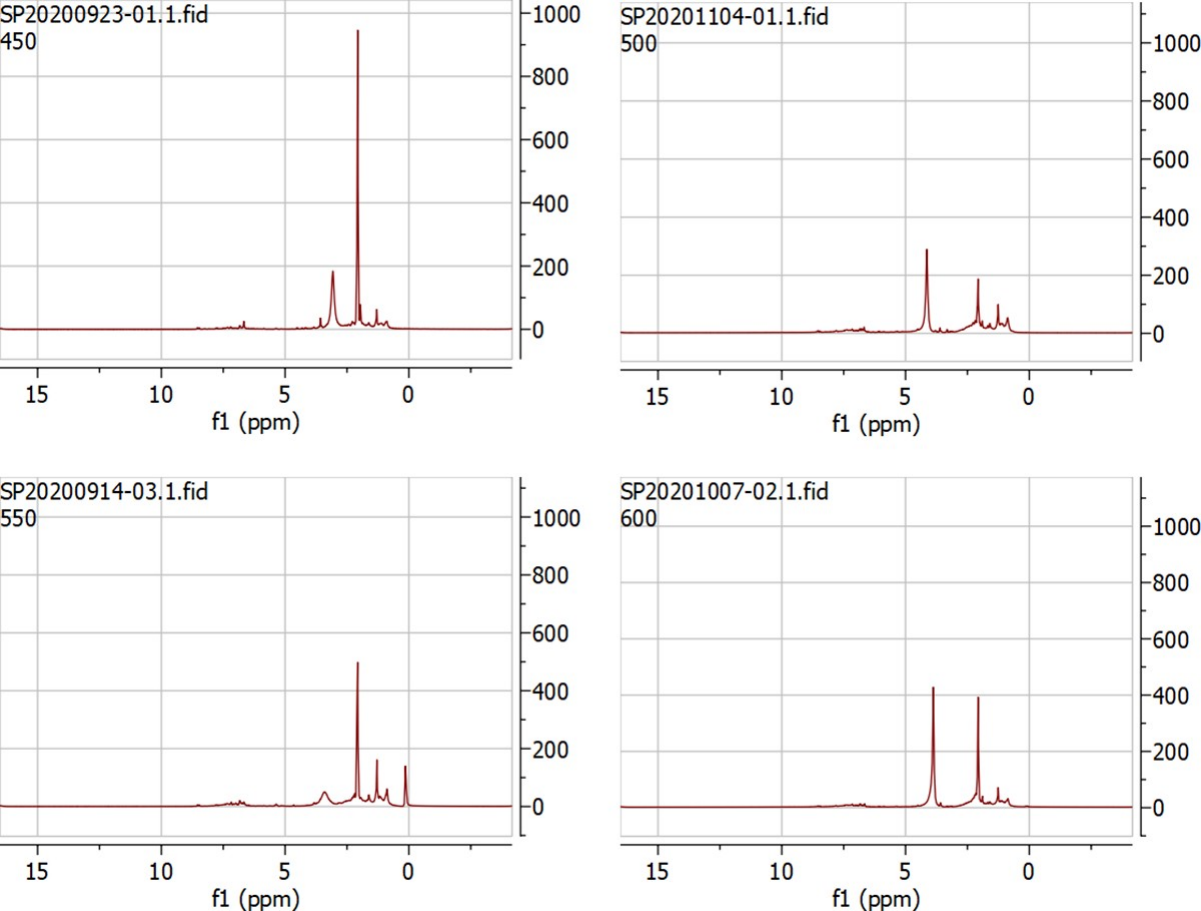
^1^H NMR spectra of the organic oils obtained in different temperature (a) 450°C, (b) 500°C, (c) 550°C and (d) 600°C.

NMR analysis was conducted on the oil samples obtained from pyrolysis temperatures of 450, 500, 550, and 600°C. The chemical shift region was identified using by ^1^H protons, and carbon assignments were based on previous reports in the literature [32,33]. The advantage of NMR analysis is that the whole bio-oil sample can be dissolved in a suitable solvent, and a quantitative assessment of the chemical functional groups can be determined by integrating the defined regions of spectra [34]. The ^1^H NMR results are shown in Fig 4 and Table 2. Overall, 1.5–3.0 ppm dominated in oils from all temperatures. Especially in the sample generated at 450°C, the highest area percentage was evident, relating to the aliphatic OH and ketone groups. Increasing temperatures led to lower percentages in the 1.5–3.0 ppm region. At 500°C, alkane and aromatic groups were found to be a major part of the oil (31.15% and 13.09%) in the regions of 0.5–1.5 and 6.0–8.5 ppm, respectively. For 550 and 600°C, the alkanes and aliphatic O-H groups, as well as ketone groups, were found to decrease with increasing temperature, whereas alcohol and methylene groups were found to behave in opposite trend.

**Table 2:** ^1^H NMR results for oil generate at different pyrolytic temperatures (in area %).

Region ppm	450°C	500°C	550°C	600°C	Type of proton
0.5–1.5	11.64	31.15	21.45	13.50	alkane
1.5–3.0	72.24	54.42	44.84	32.44	aliphatic OH, ketone
3.0–4.4	8.23	1.34	19.48	37.68	alcohol, methylene
4.4–6.0	1.58	-	3.18	5.02	methoxy, carbohydrate
6.0–8.5	6.29	13.09	10.80	11.35	(hetero-)aromatic
9.510.1	-	-	0.24	-	aldehyde

**Fig 5:**
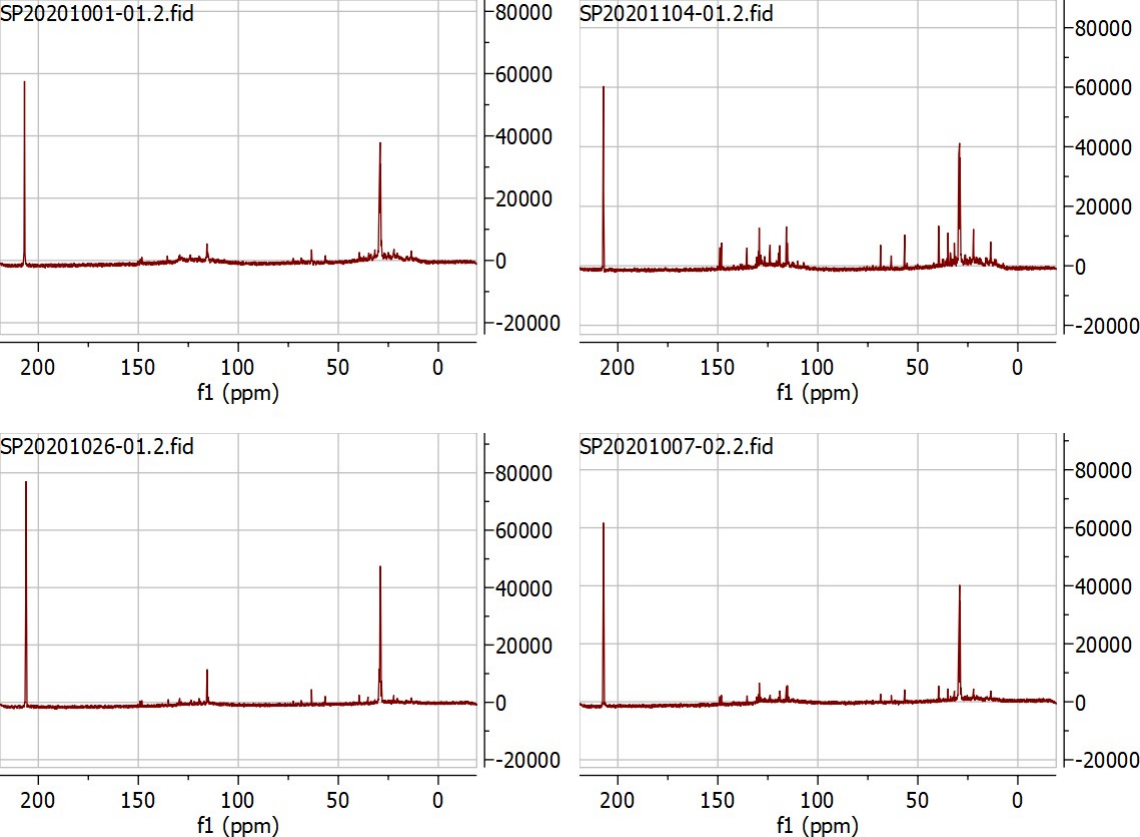
^13^C NMR spectra of the organic oils obtained in different temperature (a) 450°C, (b) 500°C, (c) 550°C and (d) 600°C.

An overview of the ^13^C NMR results is shown in Fig 5, and the percentage within a given chemical shift range is summarized in Table 3. Information on the type of chemical functional groups in the oils is provided. The region of 1–55 ppm corresponds to alkyl hydrocarbons considered for the provision of energy. Alkyl hydrocarbons are of prime interest when the bio-oil is to be used as fuel [35]. From Table 3, it can be seen that there were two significant groups which include aliphatic and aromatic compounds. The 1–28 ppm region representing short aliphatic chains was most abundant at 600°C. Higher heat was likely needed to cleave a long chain of polymer from the lignocellulosic biomass into short chain products. It is generally accepted that the short chain aliphatics, such as the alkanes (C_8_ –C_13_), are important for producing jet and diesel fuels [36]. However, high molecular weight aliphatics in the region of 28–55 ppm were obtained at 500 and 450°C, with the area percentages of about 68% and 53%, respectively. The region between 95 and 165 ppm represents aromatics, including heteroaromatics, for example, furans and alkenes in the bio-oil. The aromatic content is important for synthetic modification [35]. Carbons corresponding to the aromatic region were abundant at 550°C, accounting for about 26% of the peak area.

**Table 3:** ^13^C NMR results for oil generated at different pyrolytic temperatures (in area %).

Region ppm	450°C	500°C	550°C	600°C	Type of proton
0–28	33.67	11.99	24.59	43.01	short aliphatic
28–55	53.19	68.22	41.54	47.65	long and branched aliphatic
55–95	-	-	8.28	-	alcohol, ether, phenolic-methoxy, carbohydrates
95–165	13.14	14.87	25.58	9.33	aromatic, olefin
165–180	-	-	-	-	ester, carboxylic acid
180–215	-	-	-	-	ketone, aldehyde

#### 3.3.2 GCxGC/TOF-MS analysis

Table 4 shows the results of GCxGC/TOF-MS analysis of oils generated at different pyrolytic temperatures in terms of various organic compounds. The yield of components in the liquid product is expected to vary with temperature because the heat fragments chemical linkages in the biomass to release compounds in the liquid product. By comparing the peak area and the relative abundance in terms of area percentage, the identified bio-oil components were classified into the following main chemical groups: alkanes, alkenes, acids, furans, alcohols, phenolic compounds, nitrogen-containing compounds, benzene derivatives, ketones, and PAHs.

**Table 4:** Distribution of chemical compounds in pyrolytic bio-oil by GCxGC/TOF-MS area percentage.

Compound	450°C	500°C	550°C	600°C
**Alkanes**	**20.529**	**11.504**	**17.215**	**26.928**
Butane			0.465	
Octane	0.499		0.379	0.802
Heptane, 2, 5-dimethyl	3.266	1.723	2.35	4.224
Decane	3.995	2.422	3.195	6.854
Dodecane	5.609	3.518	4.704	
Tetradecane	2.956	1.191	2.654	5.914
Hexadecane		1.405		4.612
Heptadecane	2.284		2.014	0.212
Heneicosane	1.634	1.031	1.074	3.66
Heptacosane	0.286	0.214	0.38	0.65
**Alkenes**	**0.123**	**2.29**	**0.197**	
Limonene	0.123	0.66	0.197	
1,4-cyclohexadiene,1-methyl-		1.63		
**Acids**	**5.547**	**4.996**	**5.858**	**2.861**
Propanoic acid	2.997	0.863	1.455	2.861
Phosphonic acid, (p-hydroxyphenyl)-	2.55	4.133	4.403	
**Furans**	**2.391**	**1.719**	**1.585**	**0.359**
Furfural	0.678	0.766	1.044	
Furan,2,5-dimethyl-	0.87	0.953	0.424	0.183
Furan,2-ethyl-5-methyl-	0.228		0.117	
Furanmethanol	0.307			
Tetrahydrofuran,2,2-dimethyl-	0.308			0.176
**Alcohols**	**8.98**	**1.311**		**0.954**
Butanol	1.311	0.382		0.954
2-pentanone, 4-hydroxy-4-methyl-	7.669			
**Phenols**	**3.843**	**7.782**	**7.769**	**10.624**
Phenol				3.581
Phenol,2-methyl-	0.525	1.007	1.091	1.061
p-Cresol	0.693	1.762	1.893	1.476
Phenol,2-methoxy	0.329	0.783	0.407	0.38
Phenol,2,3-dimethyl	0.409	0.701	0.422	0.725
Phenol,4-ethyl	0.919	1.502	1.874	1.77
Phenol,2-ethyl-6-methyl-	0.191	0.788	0.586	0.597
Phenol,3,4-dimethyl-	0.062	0.165	0.182	0.123
o-Creosol	0.028			
Phenol,4-ethyl-3-methyl-	0.164		0.367	0.377
Hydroquinone	0.523	0.856	0.947	0.534
Catechol		0.218		
**N-containing**	**10.133**	**15.811**	**13.558**	**8.972**
Butanenitrile	0.704	0.867	1.303	
Pyrrole	3.566	4.215	3.91	2.623
1H-pyrrole, 3-methyl	1.154	1.963	0.806	1.741
1H-pyrrole, 1-methyl	0.789	0.849		
Indole	0.398	1.05	0.702	0.759
Pyridine,3-(1-methyl-2-pyrrolidinyl)’(s)	3.522	5.37	4.663	3.849
2H-Imidazole,2,24,5-tetramethyl-		1.497		
Pyridine			1.022	
2-propanamine,N-(1-methylethylidene)			1.152	
**Benzene derivatives**	**15.95**	**24.965**	**18.416**	**21.069**
Toluene	9.017	14.327	11.052	10.441
Ethylbenzene	2.693	3.501	2.478	3.262
O-xylene	1.739	0.988	0.923	1.059
P-xylene	0.509	2.779		3.155
Styrene	1.35	1.947		1.164
Benzene, propyl	0.298	0.539	0.368	0.367
Benzene,1-ethyl-4-methyl-	0.344	0.884	0.694	0.81
Benzene,1,3-dimethyl			2.504	
Mesitylene			0.442	
Benzene,1,2,3-trimethyl				0.811
**Ketones**	**10.482**	**7.939**	**11.159**	**6.31**
3-Pentanone	0.964	1.337	1.629	0.645
Methyl Isobutyl ketone	5.672		0.71	1.563
Cyclopentanone	1.308	0.713	0.967	1.44
2-Cyclopentane-1-one	0.834	1.42	2.741	
2-Cyclopentane-1-one, 2-methyl	0.858	1.428	1.64	0.897
2-Cyclopentane-1-one, 3-methyl		0.736	1.004	0.482
2-Cyclopentane-1-one, 2,3-dimethyl	0.846	0.741	0.949	0.775
2-Cyclopentane-1-one, 3,4-dimethyl		0.57	1.222	0.508
1,2-ethanediol,diacetate		0.994	0.297	

The alkanes and the benzene derivatives were the majority of the compounds identified for all temperatures. These alkanes were not found via GC-MS in our previous work [37]. At 600°C, the alkanes had the highest percentage of peak area, while at 500°C, the benzene derivatives were the highest. Within the alkanes, decane, tetradecane, hexadecane, and dodecane were among the most abundant components obtained at 450, 500, and 550°C, whereas toluene was predominant among the benzene derivatives, especially at 500°C. Compared to those reported in other works, this organic liquid had a high heating value [38,39]. This was because it consisted predominantly of long chain aliphatic and aromatic groups. In addition, it contained small amounts of oxygenated compounds such as ketones, phenols, acids, and furans, as well as alcohols that reduced the heating value.

The phenolic compounds in the pyrolytic oils are a typical product from lignocellulosic biomass, mainly produced from the decomposition of lignin [40,41]. They have been utilized in various household, medical, and solvent products [42]. In this work, higher temperatures tended to produce more phenolic compounds. For example, from 450 to 600°C, there was a significant increase in the percentage area of phenolics from 3.8% to 10.6%. Ketones are carbonyl compounds used mainly as solvents and intermediates in the chemical industry [43]. The results of the GCxGC/TOF-MS analysis show that a high concentration of the ketone group was found in pyrolysis products at 450 and 550°C. In particular, methyl Isobutyl ketone was the main component at 450°C, reaching 5.7%. The furan structure contains a heterocycle of four carbon atoms and one oxygen atom; it primarily results from hemicellulose decomposition and is probably produced from hexoses [44,45]. The furan yield, including furfural decreased with increasing temperature similarly [46]. This trend was opposite to the alkane yield, which suggests a hypothesis to support the possible pathway of the formation of alkanes.

Pyrrole and pyridine,3-(1-methyl-2-pyrrolidinyl), also known as nicotine, were the main nitrogen-bearing compounds of the bio-oils. The group of nitrogen-bearing compounds increased significantly from 10.13% to 15.81% at 450 to 500°C. Increasing the temperature further resulted in a gradual decline in the concentration to 13.56% at 550°C, and approximately 9% at 600°C.

### 3.3 The possible reaction pathway of alkanes

**Fig 6:**
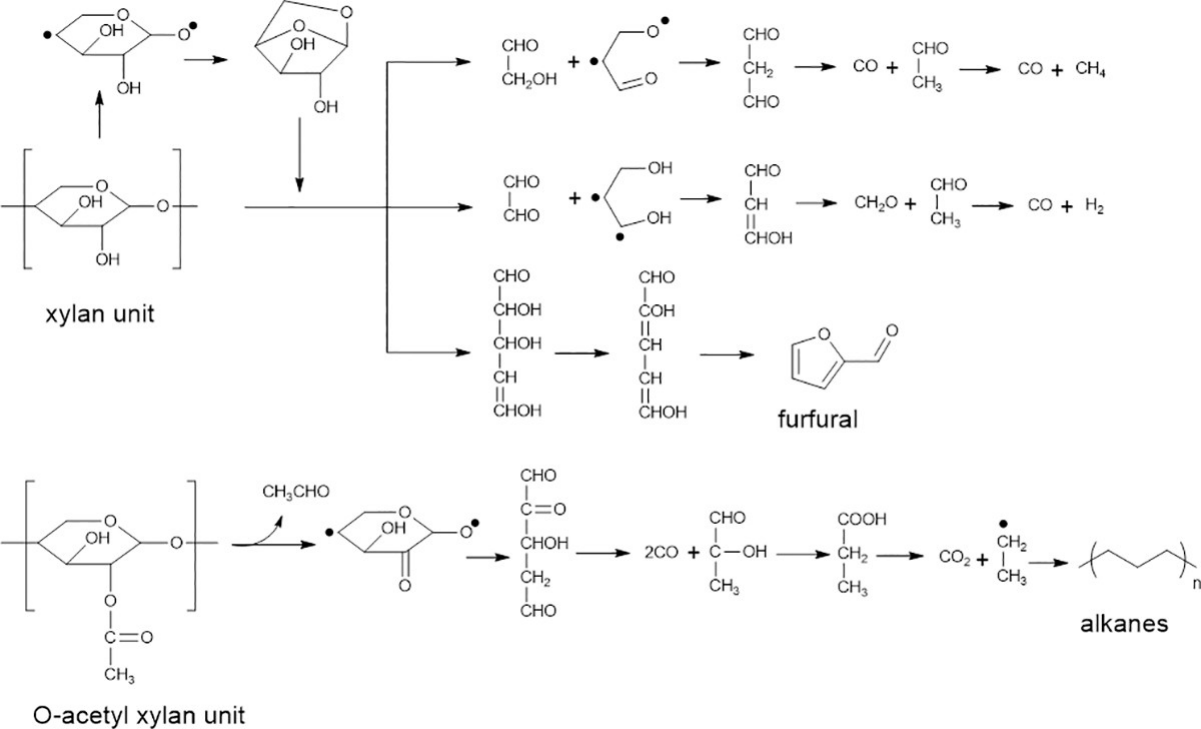
A possible pathway for conversion of hemicelluloses into alkanes, adapted from [46].

From analysis with 2-dimensional GC, alkanes were found to be rather high, based on peak area percentage. The existence of alkanes in noticeable amount found here was surprising since it was not stressed in previous published reports. It is of interest to propose a possible pathway of alkanes. Alkanes were likely formed from pyrolysis of hemicelluloses since reaction temperatures were not so high in our work. Polysaccharide chain of the hemicelluloses started with depolymerization into oligosaccharides, following the cleavage of monomeric unit including xylan and O-acetyl xylan units. The cleavage and rearrangement of both units were further decomposed to form small molecular compounds, such as furfural, CO, H_2_, CH_4_ and short alkanes as well as intermediates [46,47], as shown in Fig 6. Long chain of alkanes may have likely been formed by polymerization and recondensation of short alkanes and intermediates during low temperature quenching in a condenser.

## 4. Conclusions

In this work, vacuum ablative pyrolysis of tobacco residues was performed to generate bio-oils. The effects of reaction temperature, particle size, and rotation speed on the pyrolytic products were considered. The maximum bio-oil yield of 55% w/w was achieved at 600°C, particle size of 1 cm, and a blade speed of 10 rpm. The bio-oil obtained was of good quality with high carbon and hydrogen contents and high calorific value. The GCxGC/TOF-MS analysis indicated that the oil was comprised mainly of alkanes and benzene derivatives, which are useful components for upgrading to diesel fuel. The organic bio-oil also contained nitrogen-containing compounds such as nicotine. The ^13^C NMR analysis results confirmed the GCxGC/TOF-MS results of long aliphatic chains, the alkanes related to high carbon content. A reaction pathway for these alkanes was proposed: they were possibly converted from decomposition of hemicelluloses.

## Supporting information

S1 Fig Dispersion graphics of organic oil in different temperature (a) 450°C, (b) 500°C, (c) 550°C and (d) 600°C. (DOCX)

S2 Fig Three-dimensional diagrams of organic oil in different temperature (a) 450°C, (b) 500°C, (c) 550°C and (d) 600°C. (DOCX)

## References

[1] Chorazy T, Čáslavský J, Žvaková V, Raček J, Hlavínek P. Characteristics of pyrolysis oil as renewable source of chemical materials and alternative fuel from the sewage sludge treatment. Waste and Biomass Valorization. 2020;11(8):4491–505.

[2] Chumsawat L, Tippayawong N. Utilizing tobacco residues to generate bio-oil and biochar via ablative pyrolysis. Chem Eng Trans. 78:49–54. Available from: www.cetjournal.it.

[3] Akhtar J, Saidina Amin N. A review on operating parameters for optimum liquid oil yield in biomass pyrolysis. Renew Sustain Energy Rev. 2012;16(7):5101–9.

[4] Isahak WNRW, Hisham MWM, Yarmo MA, Yun Hin TY. A review on bio-oil production from biomass by using pyrolysis method. Renew Sustain Energy Rev. 2012;16(8):5910–23.

[5] Meier D, Faix O. State of the art of applied fast pyrolysis of lignocellulosic materials–a review. Bioresour Technol. 1999;68(1):71–77.

[6] Garcia-Nunez JA, Pelaez-Samaniego MR, Garcia-Perez ME, Fonts I, Abrego J, Westerhof RJM, et al. Historical development of pyrolysis reactors: a review. Energy Fuels. 2017;31(6):5751–5775.

[7] Luo G, Chandler DS, Anjos LCA, Eng RJ, Jia P, Resende FLP. Pyrolysis of whole wood chips and rods in a novel ablative reactor. Fuel. 2017;194:229–38.

[8] Wentrup C. Flash vacuum pyrolysis: techniques and reactions. Angewandte Chemie. 2017;56(47):14808–14835. doi: 10.1002/anie.201705118 28675675

[9] Min Ju Y, Cheol Oh K, Yol Lee K, Hyun Kim D. Performance analysis of a vacuum pyrolysis system. J Biosyst Eng. 2018;43(1):14–20.

[10] Sharma RK, Wooten JB, Baliga VL, Martoglio-Smith PA, Hajaligol MR. Characterization of char from the pyrolysis of tobacco. J Agric Food Chem. 2002;50(4):771–83. doi: 10.1021/jf0107398 11829644

[11] Lin Y, Yan W, Sheng K. Effect of pyrolysis conditions on the characteristics of biochar produced from a tobacco stem. Waste Manag Res. 2016;34(8):793–801. doi: 10.1177/0734242X16654977 27401160

[12] Valverde JL, Curbelo C, Mayo O, Molina CB. Pyrolysis kinetics of tobacco dust. Chem Eng Res Des. 2000;78(6):921–924.

[13] Cardoso CR, Miranda MR, Santos KG, Ataíde CH. Determination of kinetic parameters and analytical pyrolysis of tobacco waste and sorghum bagasse. J Anal Appl Pyrolysis. 2011;92(2):392–400.

[14] Yıldız Z, Ceylan S. Pyrolysis of tobacco factory waste biomass: TG-FTIR analysis, kinetic study and bio-oil characterization. J Therm Anal Calorim. 2019;136(2):783–94.

[15] Strezov V, Popovic E, Filkoski R V., Shah P, Evans T. Assessment of the thermal processing behavior of tobacco waste. Energy and Fuels. 2012;26(9):5930–5.

[16] Pütün AE, Önal E, Uzun BB, Özbay N. Comparison between the “slow” and “fast” pyrolysis of tobacco residue. Ind Crops Prod. 2007;26(3):307–14.

[17] Gozan M, Yasman, Wulan PPDK, Dawitri E. Tobacco leaves pyrolysis for repellent active compound production. Int J Appl Eng Res. 2014;9(21):9739–50.

[18] Cardoso CR, Ataíde CH. Analytical pyrolysis of tobacco residue: Effect of temperature and inorganic additives. J Anal Appl Pyrolysis. 2013;99:49–57.

[19] Mitsui K, David F, Dumont E, Ochiai N, Tamura H, Sandra P. LC fractionation followed by pyrolysis GC-MS for the in-depth study of aroma compounds formed during tobacco combustion. J Anal Appl Pyrolysis. 2015;116:68–74.

[20] Liu B, Li YM, Bin Wu S, Li YH, Deng SS, Xia ZL. Pyrolysis characteristic of tobacco stem studied by Py- GC/MS, TG-FTIR, and TG-MS. BioResources. 2013;8(1):220–30.

[21] Yan B, Zhang S, Chen W, Cai Q. Pyrolysis of tobacco wastes for bio-oil with aroma compounds. J Anal Appl Pyrolysis. 2018;248–54.

[22] Chen H, Lin G, Chen Y, Chen W, Yang H. Biomass Pyrolytic Polygeneration of Tobacco Waste: Product Characteristics and Nitrogen Transformation. Energy and Fuels. 2016;30(3):1579–88.

[23] Hossain MM, Scott IM, Berruti F, Briens C. Optimizing pyrolysis reactor operating conditions to increase nicotine recovery from tobacco leaves. J Anal Appl Pyrolysis. 2015;112:80–7.

[24] Xia Q, Yan B, Wang H, Xu J, Zhang S, Zhou G, et al. Production of bio-oils enriched with aroma compounds from tobacco waste fast pyrolysis in a fluidized bed reactor. Biomass Convers Biorefinery. 2020;

[25] McGrath TE, Brown AP, Meruva NK, Chan WG. Phenolic compound formation from the low temperature pyrolysis of tobacco. J Anal Appl Pyrolysis. 2009;84(2):170–8.

[26] Kibet JK, Khachatryan L, Dellinger B. Phenols from pyrolysis and co-pyrolysis of tobacco biomass components. Chemosphere. 2015;138:259–65. doi: 10.1016/j.chemosphere.2015.06.003 26091866

[27] Onorevoli B, Machado ME, Polidoro ADS, Corbelini VA, Caramão EB, Jacques RA. Pyrolysis of residual tobacco seeds: Characterization of nitrogen compounds in bio-oil using comprehensive two-dimensional gas chromatography with mass spectrometry detection. Energy and Fuels. 2017;31(9):9402–7.

[28] Garca-Pèrez M, Chaala A, Roy C. Vacuum pyrolysis of sugarcane bagasse. J Anal Appl Pyrolysis. 2002;65(2):111–36.

[29] Ertaş M, Hakki Alma M. Pyrolysis of laurel (Laurus nobilis L.) extraction residues in a fixed-bed reactor: Characterization of bio-oil and bio-char. J Anal Appl Pyrolysis. 2010;88(1):22–9.

[30] Khuenkaeo N, Tippayawong N. Production and characterization of bio-oil and biochar from ablative pyrolysis of lignocellulosic biomass residues. Chem Eng Commun. 2020;207(2):153–60.

[31] Akalina MK, Karagöz S. Pyrolysis of tobacco residue. Part 2: Catalytic. BioResources. 2011;6(2):1773–805.

[32] Ingram L, Mohan D, Bricka M, Steele P, Strobel D, Crocker D, et al. Pyrolysis of wood and bark in an auger reactor: Physical properties and chemical analysis of the produced bio-oils. Energy and Fuels. 2008;22(1):614–25.

[33] Mullen CA, Strahan GD, Boateng AA. Characterization of various fast-pyrolysis bio-oils by NMR spectroscopy. Energy and Fuels. 2009;23(5):2707–18.

[34] Joseph J, Baker C, Mukkamala S, Beis SH, Wheeler MC, Desisto WJ, et al. Chemical shifts and lifetimes for nuclear magnetic resonance (NMR) analysis of biofuels. Energy and Fuels. 2010;24(9):5153–62.

[35] Negahdar L, Gonzalez-Quiroga A, Otyuskaya D, Toraman HE, Liu L, Jastrzebski JTBH, et al. Characterization and Comparison of Fast Pyrolysis Bio-oils from Pinewood, Rapeseed Cake, and Wheat Straw Using 13C NMR and Comprehensive GC × GC. ACS Sustain Chem Eng. 2016;4(9):4974–85. doi: 10.1021/acssuschemeng.6b01329 27668136 PMC5027642

[36] Xing R, Subrahmanyam AV., Olcay H, Qi W, van Walsum GP, Pendse H, et al. Production of jet and diesel fuel range alkanes from waste hemicellulose-derived aqueous solutions. Green Chem. 2010;12(11):1933–46.

[37] Khuenkaeo N, MacQueen B, Onsree T, Daiya S, Tippayawong N, Lauterbach J. Bio-oils from vacuum ablative pyrolysis of torrefied tobacco residues. RSC Adv. 2020;10(58):34986–95. 10.1039/d0ra06014c PMC9056820 35515664

[38] Jahirul MI, Rasul MG, Chowdhury AA, Ashwath N. Biofuels production through biomass pyrolysis- A technological review. Energies. 2012;5(12):4952–5001.

[39] Haarlemmer G, Guizani C, Anouti S, Déniel M, Roubaud A, Valin S. Analysis and comparison of bio-oils obtained by hydrothermal liquefaction and fast pyrolysis of beech wood. Fuel. 2016;174:180–8.

[40] Zhang J, Choi YS, Yoo CG, Kim TH, Brown RC, Shanks BH. Cellulose-hemicellulose and cellulose-lignin interactions during fast pyrolysis. ACS Sustain Chem Eng. 2015;3(2):293–301.

[41] Figueirêdo MB, Venderbosch RH, Heeres HJ, Deuss PJ. In-depth structural characterization of the lignin fraction of a pine-derived pyrolysis oil. J Anal Appl Pyrolysis. 2020;149:104837.

[42] Bu Q, Lei H, Ren S, Wang L, Zhang Q, Tang J, et al. Production of phenols and biofuels by catalytic microwave pyrolysis of lignocellulosic biomass. Bioresour Technol. 2012;108:274–9. doi: 10.1016/j.biortech.2011.12.125 22261662

[43] Moldoveanu SC. Chapter 15 Pyrolysis of Aldehydes and Ketones. Tech Instrum Anal Chem. 2010;28:397–418.

[44] Collard FX, Blin J. A review on pyrolysis of biomass constituents: Mechanisms and composition of the products obtained from the conversion of cellulose, hemicelluloses and lignin. Renew Sustain Energy Rev. 2014;38:594–608.

[45] Iroegbu AO, Sadiku ER, Ray SS, Hamam Y. Sustainable chemicals: A brief survey of the furans. Chem Africa. 2020;3(3):481–96.

[46] Shen DK, Gu S, Bridgwater A V. Study on the pyrolytic behaviour of xylan-based hemicellulose using TG-FTIR and Py-GC-FTIR. J Anal Appl Pyrolysis. 2010;87(2):199–206.

[47] Liu WJ, Li WW, Jiang H, Yu HQ. Fates of chemical elements in biomass during Its pyrolysis. Chem Rev. 2017;117(9):6367–98. doi: 10.1021/acs.chemrev.6b00647 28337906

