# Supplementary material for: Production and characterization of bio-oils from fast pyrolysis of tobacco processing wastes in an ablative reactor under vacuum
Source: PLoS One. 2021 Jul 16;16(7):e0254485. doi: 10.1371/journal.pone.0254485 (PMC8284650; doi:10.1371/journal.pone.0254485)
Supplement: S2 Fig — (DOCX) [file pone.0254485.s002.docx]

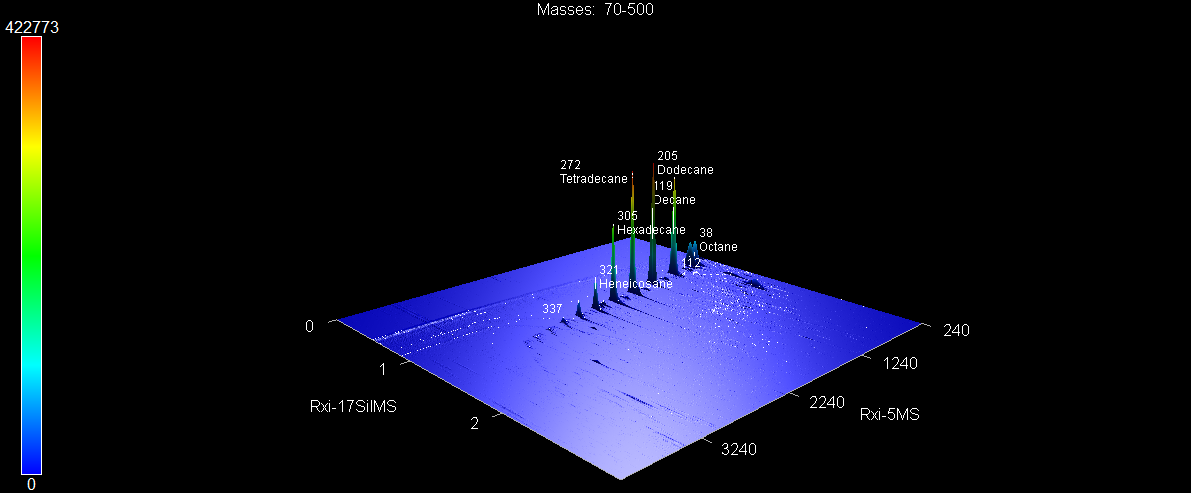


(a)


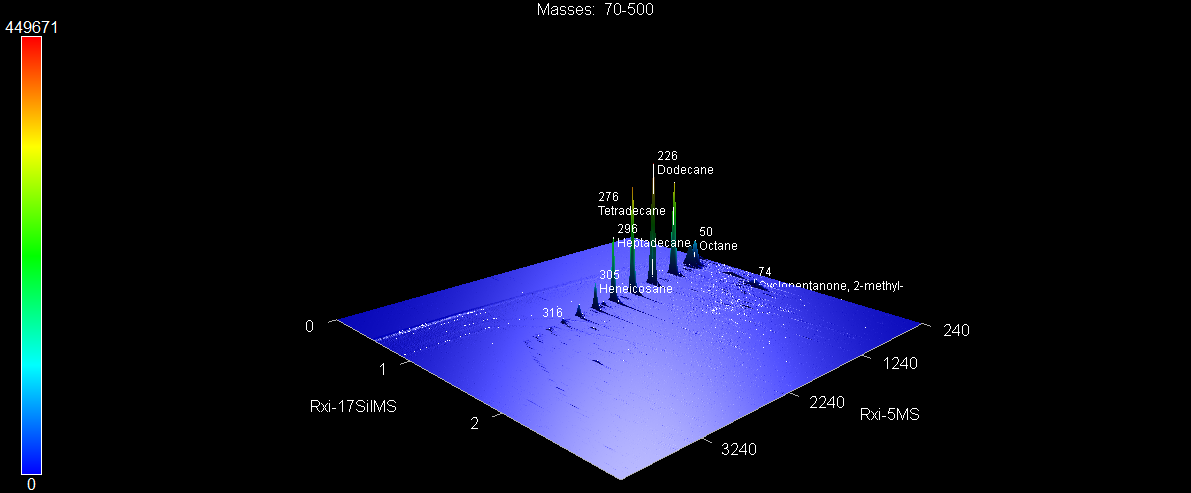


(b)


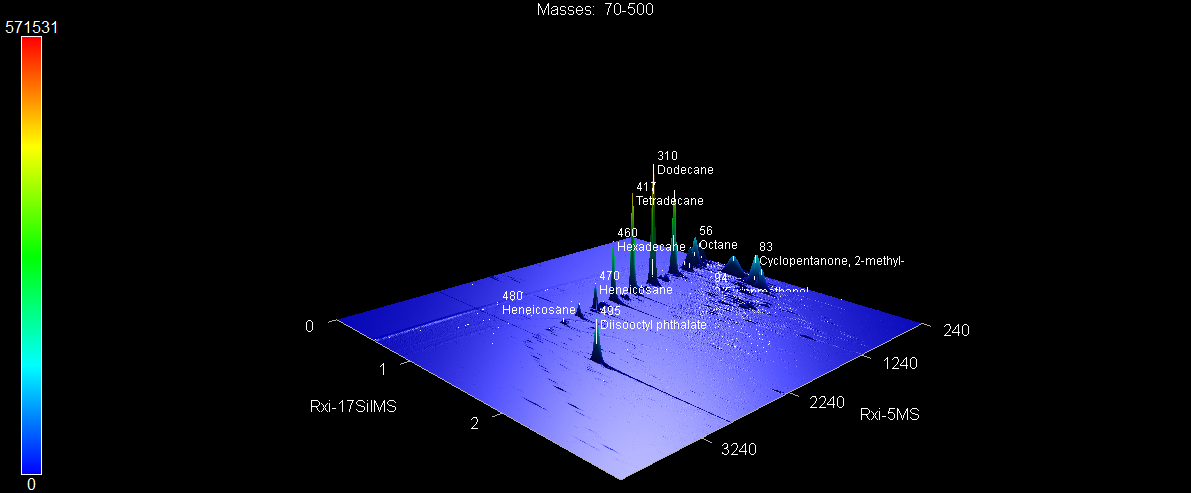


(c)


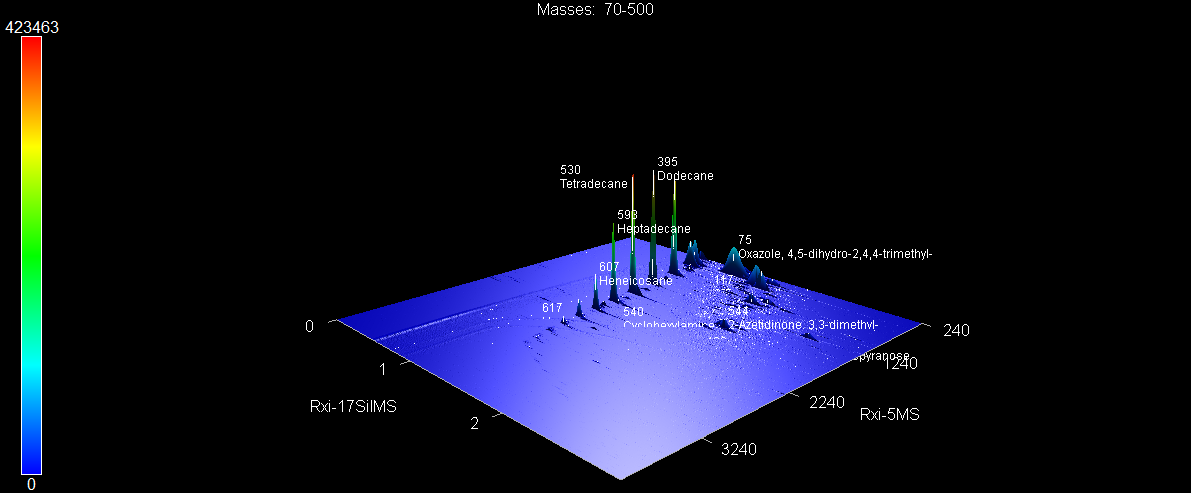


(d)

**Fig. S2** Three-dimensional diagrams of organic oil in different temperature (a) 450 ^o^ C, (b) 500 ^o^ C, (c) 550 ^o^ C and (d) 600 ^o^ C
