# Supplementary material for: Production and characterization of bio-oils from fast pyrolysis of tobacco processing wastes in an ablative reactor under vacuum
Source: PLoS One. 2021 Jul 16;16(7):e0254485. doi: 10.1371/journal.pone.0254485 (PMC8284650; doi:10.1371/journal.pone.0254485)
Supplement: Attachment — Submitted filename: Manuscript review PONE-D-21-08473.docx [file pone.0254485.s003.docx]

Manuscript review

Manuscript number: PONE-D-21-08473

Manuscript title: Yields and characterization of bio-oils from fast pyrolysis of tobacco processing wastes in an ablative reactor under vacuum

Summary:

Vacuum pyrolysis of tobacco residues was performed at various temperatures (400-600 degC) and ablative blade rotation speeds. Product analysis is reported with surprising quantities of alkanes.

General comments:

The paper has many standard experimental analyses seen in pyrolysis literature and there are some surprising results showing alkanes- though without a mass balance it is not clear how much. The biggest weaknesses of the paper are the following:

1) There is no clear hypothesis.
2) Relevant literature is not cited – notably, there are many papers out there regarding vacuum pyrolysis. Further, have other researchers found alkanes to be present in tobacco pyrolysis oils? The manuscript does not discuss if this is a new finding or not.

| Location | Comment |
| --- | --- |
| Introduction | The premise of the paper is vacuum pyrolysis, which is a very specialized technology. However you did not cite a vast body of research which has explored how vacuum pyrolysis changes the product spectrum. Please add a paragraph explaining this choice. |
| Introduction | What is the scientific question and hypothesis? Stating a list of experiments is not a very good story, so to speak. |
| Experimental procedure | Please provide a figure illustrating your unique reactor configuration. |
| Experimental procedure | It is so good that you ran the experiments 3x. Nice job. |
| Results | Please compare your results to at least some of the many, many vacuum pyrolysis papers available in the literature. E.g. Garcia-Perez et al. 2002. |
| Table 1 | Please report the ash content and proximate analysis of the char produced from this work. I am very curious what char looks like from this feedstock under vacuum pyrolysis. |
| 3.3.1 NMR analysis | Where are your spectra? Tables 2 and 3 should be connected to a figure with overlayed data. |
| Table 4 | Area percentage is a good start. Do you have an idea of mass balance from this reactor? GCxGC only captures monomer and dimer compounds, but it is well known that vacuum pyrolysis produces a large amount of lignin and sugar derived oligomers, as seen in the literature. |
| Fig. 4 | This reaction scheme does not seem likely. Hydrogenation needs either a metal-acid catalyst or very high temperatures. All of the hydrocarbons come from xylose? |
| General | What is the maximum temperature of the hot points in the reactor? |
